# Balancing Herbal Medicine and Functional Food for Prevention and Treatment of Cardiometabolic Diseases through Modulating Gut Microbiota

**DOI:** 10.3389/fmicb.2017.02146

**Published:** 2017-11-08

**Authors:** Ming Lyu, Yue-fei Wang, Guan-wei Fan, Xiao-ying Wang, Shuang-yong Xu, Yan Zhu

**Affiliations:** ^1^Tianjin State Key Laboratory of Modern Chinese Medicine, Tianjin University of Traditional Chinese Medicine, Tianjin, China; ^2^Research and Development Center of TCM, Tianjin International Joint Academy of Biotechnology & Medicine, Tianjin, China; ^3^Medical Experiment Center, First Teaching Hospital of Tianjin University of Traditional Chinese Medicine, Tianjin, China; ^4^Neuroscience Program, Neuroprotection Research Laboratory, Department of Neurology and Radiology, Massachusetts General Hospital, Harvard Medical School, Boston, MA, United States; ^5^New England Biolabs, Inc., Ipswich, MA, United States

**Keywords:** herbal medicine, functional food, cardiovascular disease, metabolic disease, intestinal microbiota

## Abstract

It has become apparent that gut microbiota is closely associated with cardiometabolic diseases (CMDs), and alteration in microbiome compositions is also linked to the host environment. Next generation sequencing (NGS) has facilitated in-depth studies on the effects of herbal medicine and functional food on gut microbiota. Both herbal medicine and functional food contain fiber, polyphenols and polysaccharides, exerting prebiotics-like activities in the prevention and treatment of CMDs. The administrations of herbal medicine and functional food lead to increased the abundance of phylum Bacteroidetes, and genus *Akkermansia, Bifidobacteria, Lactobacillus, Bacteroides* and *Prevotella*, while reducing phylum Firmicutes and Firmicutes/Bacteroidetes ratio in gut. Both herbal medicine and functional food interact with gut microbiome and alter the microbial metabolites including short-chain fatty acids (SCFAs), bile acids (BAs) and lipopolysaccharides (LPS), which are now correlated with metabolic diseases such as type 2 diabetes (T2D), obesity and non-alcoholic fatty liver disease (NAFLD). In addition, trimethylamine (TMA)-N-oxide (TMAO) is recently linked to atherosclerosis (AS) and cardiovascular disease (CVD) risks. Moreover, gut-organs axes may serve as the potential strategy for treating CMDs with the intervention of herbal medicine and functional food. In summary, a balance between herbal medicine and functional food rich in fiber, polyphenols and polysaccharides plays a vital role in modulating gut microbiota (phylum Bacteroidetes, Firmicutes and Firmicutes/Bacteroidetes ratio, and genus *Akkermansia, Bifidobacteria, Lactobacillus, Bacteroides* and *Prevotella*) through SCFAs, BAs, LPS and TMAO signaling regarding CMDs. Targeting gut-organs axes may serve as a new therapeutic strategy for CMDs by herbal medicine and functional food in the future. This review aims to summarize the balance between herbal medicine and functional food utilized for the prevention and treatment of CMDs through modulating gut microbiota.

## Introduction

The Human Microbiome Project funded by National Institutes of Health (NIH) (Qin et al., 2010) and Metagenomics of the Human Intestinal Tract (MetaHIT) consortium funded by European Commission (Turnbaugh et al., 2007) have promoted better understanding of the functional properties and healthy composition of gut microbiota. Various microbial communities and their genes (the microbiome) are present in human body, influencing human health and diseases (Human Microbiome Project, 2012). The human gut microbiota contains a diverse array of microorganisms, including bacteria, archaea and fungi that colonize the surfaces of the gastrointestinal (GI) tract; bacteriophage are also in high abundance in GI tract (Savage, 1977). Six bacterial phyla dominate the gut microbiota of healthy adult subjects: Firmicutes, Proteobacteria, Bacteroidetes, Fusobacteria, Actinobacteria, and Verrucomicrobia. The intestine hosts >10^14^ microorganisms with critical physiological roles, and the microbial compositions differ along the digestive tract (Aron-Wisnewsky and Clement, 2016). The large intestine, particularly the colon, harbors a complex and dynamic microbial ecosystem with high densities of living bacteria. These bacteria achieve concentrations of approximately 10^11^–10^12^ cells/g of luminal contents (Simon and Gorbach, 1984; Guarner and Malagelada, 2003).

A multitude of literature supports the role of gut microbiota in the development and progression of cardiometabolic diseases (CMDs). CMDs have become a worldwide epidemic, with dramatically increasing prevalence of cardiovascular disease (CVD), obesity, type 2 diabetes (T2D), non-alcoholic fatty liver disease (NAFLD), atherosclerosis (AS), hypertension, and dyslipidemia (Hansen et al., 2015; Aron-Wisnewsky and Clement, 2016; Meyer and Bennett, 2016; Woting and Blaut, 2016; Micha et al., 2017). In the search for novel therapeutic leads, the association of gut microbiota and microbial metabolites with the development of CMDs holds the potential in future drug discovery (Koopen et al., 2016).

Disruption of microbial ecosystems during crucial developmental periods could affect body physiology or cause undesired negative effects. For instance, the overuse of antibiotics in early life is associated with obesity in both humans and rodents (Cho et al., 2012). Herbal medicine such as traditional Chinese medicine (TCM) can be used as an alternative strategy to modulate microbiota and for modern drug discovery. Moreover, certain food components provided benefits beyond basic nutrition, leading to the concept of functional food and nutraceuticals. Functional food offer benefits beyond basic nutrition when consumed regularly as part of a diet. Herbal medicine and functional food produce a large diversity of secondary metabolites which display a broad array of biological and pharmacological properties (Wink, 2015) and are widely accepted as high-efficiency and low toxicity “medicinal diets” which are capable of avoiding certain side-effects. In this review article, recently discovered mechanisms of herbal medicine and functional food are summarized and their contributions to prevention and treatment of CMDs through modulating microbiota are also outlined.

## Gut microbiota in CMDs

### Gut microbiota in cardiovascular diseases

In recent years, an increasing number of reseachers begin to pay their attention to the mutual effects on intestinal flora and CVDs, resulting from the new findings of gut microbe–derived metabolite trimethylamine (TMA)-N-oxide (TMAO). Gut microbiota has an intimate relationship with CVDs, including thrombosis, AS, myocardial infarction (MI) and stroke.

#### TMAO

TMAO was first identified as a contributor to CVD in a large clinical cohort of 1,876 subjects by Stanley L. Hazen team using an untargeted metabolomics platform (Wang et al., 2011). In a subsequent expansion study of 4,007 subjects undergoing elective coronary angiography indicated an association between elevated TMAO levels in plasma and increased risk for major adverse cardiovascular events (MACE) over a 3-year period in humans (Tang et al., 2013). Then, increasing clinical reports support an involvement of plasma TMAO levels in the etiology of various CVDs. For example, elevated plasma TMAO levels in patients predict a high atherosclerotic burden (Senthong et al., 2016a), long-term adverse event risk and incremental prognostic of peripheral artery disease (PAD) (Senthong et al., 2016b), higher long-term mortality risk of coronary artery disease (CAD) (Senthong et al., 2016c), and adverse clinical outcomes in heart failure (HF) (Tang et al., 2014, 2015b; Troseid et al., 2015). Higher TMAO levels provide clinical utility in risk stratification of acute coronary syndromes (ACS) (Li X. S. et al., 2017) showing a direct pro-thrombotic effect (Zhu et al., 2017), predict close association with poor prognosis of MI (Suzuki et al., 2017). A systematic review and meta-analysis reconfirmed elevated concentrations of TMAO and its precursor TMA were associated with increased risks of MACE (Heianza et al., 2017). Furthermore, gut microbiota played an obligatory role in the metabolism of TMA, eight species (*Anaerococcus hydrogenalis, Clostridium asparagiforme, Clostridium hathewayi, Clostridium sporogenes, Escherichia fergusonii, Proteus penneri, Providenciarett geri, and Edwardsiella tarda*) in two different phyla (Firmicutes and Proteobacteria) and six genera correlated with choline consumption and TMA accumulation were identified (Romano et al., 2015). Undoubtedly, TMAO had become a new biomarker in diagnosis of CVD.

The conclusions of cumulative reports on the meta-organismal metabolic pathway for TMAO production and its possible mechanisms resulting in CVD are highlighted: ➀**TMA production:** phosphatidylcholine (PC), choline, and L-carnitine, generating the precursor TMA by gut microbiota cleavage, were abundant in dietary foods such as red meat, shellfish, egg yolk and high-fat dairy products (Wang et al., 2011). Until now, either choline or L-carnitine as substrate, TMA is produced by two identified distinct microbial enzyme systems. Catalytic unit *(cutC)* and a regulatory polypeptide *(cutD)* are required for TMA production from choline (Craciun and Balskus, 2012; Craciun et al., 2014). The catalytic protein *(CntA)* and the regulatory protein *(CntB)* are involved in TMA production form L-carnitine (Zhu et al., 2014). ➁**TMA**→**TMAO:** hepatic flavin monooxygenase 3 (FMO3) expression was up-regulated by bile acids (BAs) via nuclear receptor farnesoid X receptor (FXR) activation (Bennett et al., 2013). TMA was readily absorbed and traveled through the portal circulation to the liver and was oxidized into TMAO by FMO3 (Wang et al., 2011; Bennett et al., 2013). ➂**TMAO induced or enhanced cell phenotypic changes:** elevated plasma TMAO induced endothelial dysfunction via activating reactive oxygen species (ROS)/thioredoxin-interactive protein (TXNIP)/nod-likereceptor family pyrin domain containing 3 (NLRP3) inflammasome (Sun X. et al., 2016) and impairing endothelial nitric oxide synthase (eNOS)-derived NO bioavailability (Hu et al., 2015; Li T. et al., 2017). In addition, TMAO accelerated vascular inflammation through mitogen-activated protein kinase (MAPK) and nuclear factor-κB (NF-κB) signaling (Seldin et al., 2016), reduced endothelial self-repair, and increased monocyte adhesion partly via the pathway of protein kinase C (PKC)/NF-κB/vascular cell adhesion molecule-1 (VCAM-1) activation (Ma et al., 2017). Moreover, TMAO contributed to macrophage cholesterol accumulation and foam cell formation (Wang et al., 2011). Furthermore, TMAO elevated platelet hyperreactivity, enhancing agonists-induced platelet activation through intracellular Ca^2+^ mobilization (Zhu et al., 2016). These changes in cell phenotype contribute to atherosclerotic CVD. ➃**TMAO promoted CAD in animal studies:** TMAO accelerated AS by reversing cholesterol transport and altering bile acids (BAs) composition (Koeth et al., 2013), enhanced thrombosis formation by activating platelet (Zhu et al., 2016), exacerbated pressure overload–induced heart failure by inducting adverse cardiac remodeling (Organ et al., 2016). Attention should be paid to the gender identity in the study of TMAO synthesis, since FMO3 expression is higher in females than males in both human and mouse (Bennett et al., 2013).

For further research, pharmacologic inhibition of TMAO producition will be a potential therapeutic strategy to reduce CVD events by targeting microbial community, microbial enzyme and/or FMO3 expression. The mechanisms of TMAO pathway (PC, choline, L-carnitine→TMA→FMO3→TMAO) linking the specific microbiota to cardiovascular functionis are very important and need to be further elucidated. In addition to the new finding of TMAO, other microbial metobolites such as SCFAs (Marques et al., 2017), BAs (Mayerhofer et al., 2017), and LPS (Pastori et al., 2017) which are beneficial for CVD will not be discussed in details here.

### Gut microbiota in metabolic diseases

A considerable number of publications have reported correlations between gut microbiota and metabolic diseases (Moreno-Indias et al., 2014; Janssen and Kersten, 2015; Greenhill, 2016; Saad et al., 2016; Sonnenburg and Backhed, 2016; Woting and Blaut, 2016). Specific metabolic abnormalities such as pro-inflammatory states, insulin resistance, glucose intolerance, dyslipidemia, high blood pressure and NAFLD, which accompanies gut microbiota dysbiosis, often develop in obese people. Moreover, obesity and T2D are considered as a medical condition, which not only contributes to the risk of developing CVD and cancer, but also negatively affects longevity and quality of life. Here, we describe several crucial mechanisms (mainly about SCFAs, LPS and BAs) that contribute to understanding the correlation between gut microbiota and obestiy/T2D.

#### SCFAs

The intestinal microbial fermentation and degradation of dietary nondigestible fiber and polysaccharides to SCFAs (acetate, propionate and butyrate) are regarded as potential metabolic targets to prevent obesity/T2D in glucose metabolism and insulin resistance. Besides, several mechanisms correlate with SCFAs affect body weight via energy intake and energy harvesting, and link with insulin sensitivity through inflammatory resoponse, lipid storage and adipose tissue function (Canfora et al., 2015). SCFAs, serving as energy substrates, directly inhibit histone deacetylases (HDACs) and activate G-protein-coupled recepotors (GPCRs). Moreover, butyrate also has effect on epithelial barrier function by increasing mucus production and protein zonula occludens-1 (ZO)-1, occludin expression (Bordin et al., 2004; Peng et al., 2007). GPR41and GPR43 targets are of significance for SCFAs. Gut microbiota promotes adiposity and body weight via SCFAs receptor GPR41. The expression of peptide YY (or PYY), a key hormone involved in the elevation of intestinal transit rate and reduction in energy hearvest, is decreased in GPR41^−/−^ mice (Samuel et al., 2008). On the contrary, SCFAs may prevent obesity via activation of GPR43. Normal diet GPR43^−/−^ mice are obese, whereas HFD-fed GPR43^+/+^ mice remain lean. Insulin signaling in adipocytes and fat accumulation in white adipose tissue (WAT) are inhibited via acetate-medatied GPR43 (Kimura et al., 2013). These distinct differences remain to be analyzed in how the gut microbiota is modulated. Besides, GPCR43 activation by SCFAs promotes the release of glucagon-like peptide-1 (GPL-1) by intestinal enteroendocrine L cells, thereby leading to insulin release and stimulating glucose tolerance (Tolhurst et al., 2012). Furthermore, a recent paper reported that acetate contributes to GPR43-mediated intestinal IgA response to microbiota, leading to crucial role in intestinal homeostasis maintenance and intestines inflammation denfence (Wu et al., 2016). Apparently, SCFAs-mediated GPCRs signaling in mice shows extensive effects on obesity/T2D, but the role of GPR41/43 signaling in humans remains to be established.

#### LPS

Endotoxin LPS is a major component of the gram-negative bacterial (such as *Escherichia coli*) outer membrane. HFD-induced gut microbiota dysbiosis can alter gut permeability and then increase cirlulating LPS levels which promotes low-grade inflammation and insulin resistance and, ultimately, obesity and T2D in rodents and humans (Cani et al., 2007, 2008; Creely et al., 2007). In addition, Increased intestinal epithelial barrier permeability is due to increased endocannabinoid system tone (Muccioli et al., 2010) and tight junctions (ZO)-1, occludin and claudin-1 expression (Wang J. H. et al., 2014). LPS stimulates inflammatory response mainly by binding to CD14/Toll-like receptor 4 (TLR4) which is respnsible for the recruitment and activation of MyD88 adaptor and NF-κB transcription factor, inducing the pro-inflammatory factors interleukin-6 (IL-6), interleukin-1β (IL-1β) and monocyte chemoattractant protein-1 (MCP-1) secretion (Hennessy et al., 2010), and therefore it triggers matabolic diseases (Robbins et al., 2014; Kang et al., 2016). Metabolic characteristics of obesity and T2D in mice were not initiated by injecting LPS when CD14/TLR4 receptor was genetically deleted, showing the significant contribution of LPS/CD14/TLR4 signaling (Shi et al., 2006; Cani et al., 2007, 2008; Poggi et al., 2007). Unexpectedly, insulin is more sensitive in TLR4^−/−^ (Shi et al., 2006) mice, but less in TLR6^−/−^ (Vijay-Kumar et al., 2010) mice with the modulator of gut microbiota than wild-type controls.

#### BAs

BAs is produced in the liver from cholesterol and metabolized in the gut by the intestinal microbiota (Midtvedt, 1974). Inversely, BAs can modulate gut microbial composition via innate immune genes activation in the small intestine (Wahlstrom et al., 2016). Cholic acid (CA) and chenodeoxycholic acid (CDCA) are the primary BAs produced in humans, whereas CA and muricholic acids (MCAs) are generated in rodents. Besides, mice also produce ursodeoxycholic acid (UDCA) as primary BAs (Sayin et al., 2013), whereas as a secondary BA in human (Ishizaki et al., 2005). The primary BAs are converted into secondary BAs by gut microbial modifications. BAs play multiple roles in the control of obesity/T2D related glucose and lipid metabolism, and energy homeostasis by activating the nuclear FXR and the cytoplasmic G protein-coupled membrane receptor 5 (TGR5) which regulate a large number metabolic pathways in the host (Thomas et al., 2008, 2009; Wahlstrom et al., 2016). On one hand, FXR is activated mainly by the CA and CDCA (Makishima et al., 1999; Parks et al., 1999; Wang et al., 1999), while TGR5 is stimulated mostly by LCA and DCA which are secondary BAs metabolized from CA and CDCA (Maruyama et al., 2002; Chen X. et al., 2011). On another, Tα/βMCA (primary BAs in mice) and UDCA inhibit FXR activation (Li et al., 2013; Sayin et al., 2013; Mueller et al., 2015). Furthermore, GLP-1 synthesis is inhibited by FXR activition (Trabelsi et al., 2015), while it is activated and secreted by TGR5 activation in colonic L cells (Thomas et al., 2009). GLP-1 signaling may be exploited into a new therapy for T2D with the help of gut microbiota (Claus, 2017; Grasset et al., 2017). Hepatic cholesterol 7a-hydroxylase (CYP7A1) is regulated by intestinal FXR with the contribution of a fibroblast growth factor15 (FGF15) activity (Inagaki et al., 2005). What's more, recent study showed HFD-fed FXR^−/−^ mice an obesity phenotype compared to the wild-type mice (Parséus et al., 2016). Thus, targeting BAs, FXR, and/or TGR5 signaling with microbiota, may shed a new light on preventing or treating metabolic diseases. At present, our knowledge on the mutual effects between BAs and gut microbiota is still far from complete.

In recent studies, the gut metabolite TMAO is also found to have an intimate relationship with metabolic diseases such as T2D (Dambrova et al., 2016; Tang et al., 2016; Schugar et al., 2017), NAFLD (Chen Y. M. et al., 2016), chronic kidney disease (CKD) (Tang et al., 2015a; Xu K. Y. et al., 2017) and bariatric surgery (Troseid et al., 2016), along with the metabolic functions including insulin resistance (Oellgaard et al., 2017) and BAs metabolism (Wilson et al., 2016). With the rapid development in the field of intestinal microbiota, the gut metabolites like TMAO, SCFAs, LPS, and BAs with their signaling interplay between microbiota, have evolved as promising avenues for prevention and treatment of CMDs. Herbal medicine and functionl food with the properity of muti-ingredient, muti-target and muti-pathway action may serve as a prebiotic-like remediation (Laparra and Sanz, 2010; Xu J. et al., 2017). How might they work in CMDs by modulating gut microbiota are discussed below.

## Herbal medicine and gut microbiota

The effectiveness of antibiotics in modern medicine has diminished somewhat due to the development of multi-drug resistant bacteria after using for more than 70 years. New classes of antimicrobial drugs are unlikely to become widely available any time soon (Laxminarayan et al., 2016). If and when they do, bacteria, viruses and other microbes will again evolve antimicrobial resistance (AMR) through variety of ways including horizontal gene transfer of mobile genetic elements (Carroll et al., 2014; Jorgensen et al., 2016). Experimental evidence, particularly rodent studies, showed convincingly that prebiotics, non-digestible, fermentable carbohydrates and fibers are capable of enhancing the growth of specific beneficial gut bacteria, thus reducing body weight, reversing insulin resistance and exerting anti-inflammatory effects (Bindels et al., 2015; Sonnenburg and Backhed, 2016). However, these effects have yet to be confirmed by intervention studies in human. Recent investigations support the idea of the involvement of intestinal bacteria in host metabolism and preventative therapeutic potential of prebiotic interventions for CMDs. Herbal medicine may therefore serve as a potential prebiotic remedy to treat CMDs and complications.

Several herbal medicine formulae, herbals and nutraceuticals that contain fiber, polyphenol, polysaccharide and certain other substances have anti-obese, anti-diabetic and anti-atherosclerotic effects through the modulation of diverse gut microbiota. These herbals with their components have the potential to be a new source for CMD drugs discovery that target specifically the gut microbiota, as summarized in Tables 1–3. According to the early direct evidence in 187 T2D patients, a herbal formula Gegen Qinlian Decoction (GQD) including four herbs: Gegen *(Radix Puerariae)*, Huangqin *(Radix Scutellariae)*, Huanglian *(RhizomaCoptidis)* and Gancao (Honey-fried *Licorice Root*), showed the anti-T2D effect partly by enriching the amounts of specific beneficial bacteria *Faecalibacterium* spp. (Xu et al., 2015). Interestingly, both *Rhizoma coptidis* (as the major component of GQD) and berberine (as the main phytochemicals of *Rhizoma coptidis*) are confimed to have an anti-obese effect by inhibiting the ratio of Firmicutes/Bacteroidetes, and lowering the growth of *Lactobacillus* (a classical type of Firmicutes) in HFD-fed mice feces. In addition, *Rhizoma coptidis* and berberine can reduce HFD-induced body and visecral adipose weights, and blood glucose and lipid levels in mice (Xie et al., 2011). What's more, berberine increases putative SCFA-producing bacteria, including *Blautia, Allobaculum, Bacteriodes, Blautia, Butyricoccus*, and *Phascolarctobacterium*, possibly leading to anti-obese and anti-diabetic effects in the host (Zhang et al., 2012a, 2015). *Rhizoma coptidis* and berberine, also the main ingredients of GQD, may contribute to the significant resistance to metabolic disease by targeting intestinal microbiota, which need to be further confirmed in clinical trials. A recent study showed that berberine improved non-alcoholic steatohepatitis (NASH) by restoring Biffidobacteria and reducing Firmicutes/Bacteroidetes ratio (Cao et al., 2016). Another herbal formula Qushi Huayu Decoction (QHD), a mixture of five herbs *(Artemisia capillaries Thunb, Gardenia jasminoides Ellis, Fallopia japonica, Curcuma longa* L., and *Hypericum japonicum Thunb.)* and two active ingredients (geniposide and chlorogenic acid) reduces oxidative stress and inflammatory response in liver by inducing glutathione-generating enzymes, decreases lipid synthesis and elevates steatosis by inhibiting glucokinase expression, and ameliorates gut barrier function and alleviates liver inflammation by inducing Treg-producing baceria. In these studies, 12 phyla of gut bacteria were altered, including increased Fusobacteria, Lentisphaerae, Verrucomicrobia, Cyanobacteria, Deferribacteres, Proteobacteria, and Bacteroidetes, as well as decreased Firmicutes, Tenericutes and Actinobacteria (Yang et al., 2017).

**Table 1:** Herbal formulae and gut microbiota.

**Formulae**	**Herbs & ingredients**	**Objects**	**Diseases**	**Physiological function related to gut microbiota**	**Gut microbiota**	**References**
Gegen Qinlian Decoction (GQD)	**Herbs:** *Radix Puerariae* (Ge Gen), *Radix Scutellariae* (Huang Qin), *Rhizoma Coptidis* (Huang Lian), *Honey-fried Licorice Root* (Gan Cao), **Ingredients:** Baicalin, Puerarin, Berberine	187 T2D patients	T2D	➀Enrich beneficial bacteria, ➁Reduce blood glucose and glycated hemoglobin.	**Increased:** *Faecalibacterium* spp.	Xu et al., 2015
Sancai Lianmei Particle (SLP)	**Herbs:** *Panax ginseng* (Ren Shen), *Rhizoma Atractylodis Macrocephalae* (Bai Zhu), *Coptis chiinensis* (Huang Lian)	60 T2D patients	T2D	Regulate intestinal flora of T2D and have similar function with acarbose.	N/A (Not Applicable)	Fang et al., 2016
Ginseng decoction	**Herbs:** *Panax ginseng* (Ren Shen), **Ingredients:** Ginseng polysaccharides, Ginsenosides	SD rat	Over-fatigue and acute cold stress model (OACS)	➀Improve intestinal metabolism and absorption of certain ginsenosides, ➁Reinstate the holistic gut microbiota.	**Increased:** *Lactobacillus* spp. *Bacteroides* spp.	Zhou et al., 2016
Daesiho-tang (Korea)	**Herbs:** *Bupleuri radix* (Chai Hu), *Pinelliae rhizome* (Ban Xia), *Zingiberis rhizome* (Gan Jiang), *Scutellariae radix* (Huang Qin), *Paeoniae radix* (Shao Yao), *Zizyphu sfructus* (Da Zao), *Ponciri fructus*, *Rheiundulati rhizome*	C57BL/6 mice	Obesity	➀Ameliorate body weight gain and body fat, ➁Regulate adiponectin and leptin genes expression, ➂Exert an anti-diabetic effect by attenuating fasting glucose level and serum insulin level, ➃Reduce TC, TG and increase HDL, GPT and GOT levels and reduce fat droplets accumulation.	**Increased:** Bacteroidetes/Firmicutes *ratio*, *Bacteroidetes*, *Lactobacillus*, *Akkermansia*, *Bifidobacterium*. **Decreased:** Firmicutes.	Hussain et al., 2016
Yupingfeng polysaccharides	**Herbs:** *Astragali radix* (Huang Qi) *Atractylodes macrocephala rhizome* (Bai Zhu), *Radix saposhnikoviae* (Fang Feng), **Ingredients:** Yupingfeng polysaccharides	Weaning rex rabbits	Immune-related diseases	➀Promote growth and immune activities, improve intestinal microbiota homeostasis and maitain intestinal barrier functionality and integrity, ➁Enhance *IL-2, IL-4, IL-10, TNF-α, TLR2, TLR4* mRNA levels; improve IL-1, IL-2, IL-4, IL-6, IL-10, IL-12, TNF-α, IFN-γ protein levels, and *TLR2, TLR4* mRNA expressions.	**Increased:** Cellulolytic bacteria **Decreased:** *Streptococcus* spp. *Enterococcus* spp.	Sun H. et al., 2016
Qushi Huayu Decoction (QHD)	**Herbs:** *Artemisia capillaries Thunb* (Yin Chenhao), *Gardenia jasminoides Ellis* (Zhi Zi), *Fallopia japonica* (Hu Zhang), *Curcuma longa L*. (Jiang Huang), *Hypericum japonicum Thunb*. (Tian Jihuang), **Ingredients:** Geniposide, chlorogenic acid.	SD rats	NAFLD	➀Decrease serum LPS, hepatic lipid synthesis, and regulatory T cell inducing microbiota, ➁Improve gut barrier function and hepatic anti-oxidative mechanism.	**Increased:** Fusobacteria, Lentisphaerae, Verrucomicrobia, Cyanobacteria, Deferribacteres, Proteobacteria, Bacteroidetes, **Decreased:** Firmicutes, Tenericutes, Actinobacteria.	Feng et al., 2017

**Table 2:** Herbs and gut microbiota.

**Herbs**	**Ingredients**	**Objects**	**Diseases**	**Physiological function related to gut microbiota**	**Gut microbiota**	**References**
*Rehmannia glutinosa Libosch* (Shu Dihuang)	N/A	Twenty 40–65 years old female middle-aged subjects with obesity	Obesity	Decrease waist circumference.	**Increased:** Actinobacteria, *Bifidobacterium*, **Decreased:** Firmicutes, *Blautia*.	Han et al., 2015
*Ganoderma lucidum* (Ling Zhi)	Polysaccharides	C57BL/6NCrlBltw mice	Obesity	➀Reverse HFD-induced gut dysbiosis, ➁Anti-obesity.	**Decreased:** Firmicutes/Bacteroidetes ratio, Proteobacteria.	Chang C. J. et al., 2015
*Flos Lonicera* (JinYinghua)	Flavonoids, Organic acids, Saponins, Iridoid glycosides	SD rats	Obesity and metabolic endotoxemia	➀Decrease body weights, ➁Lower endotoxin, aspartate transaminase, HDL, triglyceride levels, ➂Reduce lipid accumulation and alleviate urinary lactulose/mannitol ratio.	**Increased:** *Akkermansia* spp., Bacteroidetes/Firmicutes ratio.	Wang et al., 2014
*Rhizoma Coptidis* (Huang Lian)	Berberine	C57BL/6J mice	Obesity	Lower degradation of dietary polysaccharides, decrease potential calorie intake, increase Fiaf protein and its related gene expressions of mitochondrial energy metabolism.	**Increased:** Bacteroidetes/Firmicutes ratio, **Decreased:** *Lactobacillus*.	Xie et al., 2011
Lingonberry (*Vaccinium vitis-idaea L*.)	20% lingonberries	C57BL/6J mice	Obesity	➀Reduce endotoxemia and inflammation, ➁Anti-obesity.	**Increased:** *Akkermansia/Faecalibacterium* ratio.	Heyman-Linden et al., 2016
*Herba Epimdii* (Yin Yanghuo)	Icariin, Epimedin A, B, C	SD rats	Osteoporosis	Enhance epimedium flavonoids absorption and antiosteoporosis activity.	N/A	Zhou et al., 2015
*Garcinia cambogia* (Teng Huangguo)	Extract	C57BL/6J mice	Obesity	Alleviate weight gain and adiposity	**Decreased:** *Clostridium aminophilum*.	Heo et al., 2016
*Cassia obtusifolia* L. (Jue Mingzi)	Anthraquinone	SD rats	NAFLD	➀Evaluate Lipid metabolism and gut microbiota diversity, ➁Up-regulate *FXR, CYP7A1, LDL-R* mRNA and PPAR-α protein levels, down-regulate *HMGCR, PPAR-γ* and *SREBP-1c* expression.	**Increased:** *Lactobacillus*, *Bacteroides*, *Parabacteroides*, **Decreased:** *Oscillospira*.	Mei et al., 2015
*Radix ginseng rubra* (Hong Shen) and *Semen Coicis* (Yi Yiren)	N/A	Wistar rat	Ulcerative colitis (UC)	Improve gut microbiota structure and relieve the ulcerative Colitis symptom.	**Increased:** *Bifidobacterium*, *Lactobacillus*.	Guo et al., 2015
*Polygonatum kingianum* (Dian Huangjing)	Polysaccharides, Saponins	SD rats	T2D	➀Reduce SCFAs production, ➁Decrease LPS level, ➂Partial recover insulin secretion and fasting blood glucose levels	**Increased:** Family *Ruminococcaceae*, Genus *Ruminococcus*.	Yan et al., 2017
Adlay	Polyphenol extract	Wistar rats	High cholesterol-related disease	Ameliorate and LDL cholesterol restore HDL cholesterol.	**Decreased:** *Erysipelotrichales*, *Clostridia*.	Wang Q. et al., 2015

**Table 3:** Herbal phytochemicals and gut microbiota.

**Phytochemicals**	**Catergory**	**Objects**	**Diseases**	**Physiological function related to gut microbiota**	**Gut microbiota**	**References**
Resveratrol and epigallocatechin-3-gallate	Polyphenol	37 obese men and women	Obestiy	Increase fat oxidation	**Decreased:** Bacteroidetes, *Faecalibacterium* *Prausnitzii*.	Most et al., 2017
Resveratrol	Polyphenol	(1) C57BL/6J ApoE^−/−^ mice	(1) AS	(1) **AS-related** ➀Reduce TMA production → decrease TMAO synthesis in liver → inhibit AS, ➁Increase BSH activity → promote generation of unconjugated BAs. ➂Decrease BA content → inhibit FXR-FGF15 axis → increase *CYP7A1* expression → induce neosynthesis of hepatic BA → promote cholesterol homeostasis → attenuate AS.	(1) **Increased:** *Lactobacillus*, *Bifidobacterium*.	Qiao et al., 2014; Chen M. L. et al., 2016
		(2) Kunming mice	(2) Obesity	(2) **Obesity-related** ➀Decrease body and visceral adipose weights, and reduce lipid and blood glucose levels, ➁Increase *Fiaf* gene expression, and decreases *LPL, SCD1, PPAR-γ, ACC1, Fas* mRNA expression correlation with fatty acids synthesis, lipogenesis and adipogenesis.	(2) **Increased:** Bacteroidetes/Firmicutes ratio, *Lactobacillus*, *Bifidobacterium*. **Decreased:** *Enterococcus faecalis*.	
Berberine	Alkaloid	(1) Wistar rats	(1) Obesity, insulin resistance	(1) ➀Inhibit obesity and insulin resistance development, ➁Increase LPS-binding protein MCP-1, leptin levels, and decrease adiponectin level, ➂Elevate SCFA levels in the intestine.	(1) **Increased:** *Blautia, Allobaculum*.	Xie et al., 2011; Zhang et al., 2012a, 2015; Cao et al., 2016; Wang Y. et al., 2017
		(2) Wistar rats	(2) Obesity	(2) Enrich bacteria produced by SCFA and reduce microbial diversity	(2) **Decreased**: *Allobaculum*, *Bacteroides*, *Blautia*, *Butyricoccus*, *Phascolarctobacterium*	
		(3) C57BL/6J mice	(3) Obesity	(3) Decrease dietary polysaccharides degradation, lower the intake of potential calorie, and activate the expressions of Fiaf protein and related genes of mitochondrial energy metabolism.	(3) **Increased**: Bacteroidetes/Firmicutes ratio, **Decreased:** *Lactobacillus*, Bacteroidetes/Firmicutes ratio,	
		(4) BALB/C Mice	(4) NAFLD	(4) Reduce body weight, and lipids, glucose, insulin level in serums. Improve transaminase activity and NAFLD activity score through down-regulated CD14, IL-1, IL-6, TNF-α.	(4) **Increased**: Bacteroidetes/Firmicutes ratio, *Bifidobacteria*.	
		(5) SD rat	(5) Energy metabolism	(5) ➀Promote butyrate production, ➁Reduce blood lipid and glucose level, ➂Regulate energy metabolism by suppressing bacterial ATP production and nicotinamide adenine dinucleotide phosphate (NADH) levels.	(5) **Increased**: *Enterobacter*, *Escherichia–Shigella*.	
Quercetin	Polyphenol	Wistar rats	Obesity	Prevent body weight gain and reduce serum insulin levels and insulin resistance.	**Decreased:** Firmicutes/Bacteroidetes ratio, *Erysipelotrichaceae*, *Bacillus, Eubacteriumcylindroides*	Etxeberria et al., 2015a
Curcumin	Polyphenol	(1) 129/SvEv mice, germ-free Il10^−/−^ mice	(1) Colitis and colon cancer	(1) Increase survival, decrease colon weight/length ratio, eliminate tumor burden.	(1) **Increased:** *Lactobacillus*, Lactobacillales, Bifidobacteriales, Erisipelotrichales, Coriobacteriales, **Decreased:** *Clostridiales*, Firmicutes.	Ghosh et al., 2014; Mcfadden et al., 2015
		(2) LDLR^−/−^ mice	(2) AS	(2) ➀Decrease LPS levels, ➁Increase intestinal barrier function by restoring intestinal alkaline phosphatase activity and tight junction proteins ZO-1 and Claudin-1 expression, ➂Reduce glucose intolerance and AS.	(2) N/A	
*Ophiopogon japonicas* polysaccharide MDG-1	polysaccharide	C57BL /6J mice	Obesity	Improve gut microbiota diversity and promote proliferation.	**Increased:** Taiwan *lactobacillus*, *Lactobacillus murinus*	Shi et al., 2015
Pterostilbene	Polyphenol	Zucker (fa/fa) rats	Obesity	Improved metabolic function (insulin sensitivity) and Anti-obesity.	**Increased:** *Akkermansia*, *Odoribacter*, Verrucomicrobia, **Decreased:** Firmicutes.	Etxeberria et al., 2016
Rhein	Polyphenol	C57BL/6J	Obesity	➀Reduce body weight and improve glucose tolerance, ➁Inhibit macrophage accumulation, anti-neuroinflammation and improve BDNF expression.	**Increased:** *Bifidobacterium* spp. *Lactobacillus* spp. **Decreased:** *Prevotella* spp. *Desulfovibrios* spp.	Wang et al., 2016
Taurine	Amino acid	BALB/C mice	Neuroendocrine functions	Increase SCFA content in feces, decrease LPS content in serum.	**Decreased:** Proteobacteria (especially *Helicobacter*)	Yu et al., 2016

Resveratrol (RSV), a natural polyphenolic compound extrated from herbal medicine *Rhixoma Polygoni Cuspidati* or functional food peanut, grape, and *Fructus Mori*, exerts antioxidant, anti-inflammatory (Walker et al., 2014), anti-tumor, cardioprotective, aging-delay, and anti-obesity effects (Baur and Sinclair, 2006; Zhang et al., 2012b). On one hand, RSV decreases TMAO levels and increases hepatic BA neosynthesis via increasing the genera *Lactobacillus* and *Bifidobacterium*, thus attenuating TMAO-induced AS in ApoE^−/−^ mice. RSV-induced BA neosynthesis was partially mediated through the enterohepatic FXR-fibroblast growth factor 15 (FGF15) axis (Chen M. L. et al., 2016). On another, RSV increases the ratio of Bacteroidetes/Firmicutes and the growth of *Lactobacillus* and *Bifidobacterium*. It also reduces the growth of *Enterococcus faecalis* through fasting-induced adipose factor (*Fiaf*, a key gene expresses in the intestine and negatievely regulated by interstinal flora) signaling, decelerating the development of obesity (Qiao et al., 2014). RSV is probably an unique and firstly reported natural product that mediates protection against both CVD and metabolic diseases via gut microbiota to date. In addition, quercetin, a key member of the polyphenol family, is discovered in numerous medicinal botanicals, including *Ginkgo biloba, Hypericum perforatum*, and *Sambucus canadensi* and also found in a variety of functional foods including apple, grape, berry, onion and tea (Li Y. et al., 2016). Intake of quercetin reduced body weight gain and attenuated serum insulin levels by reducing Firmicutes/Bacteroidetes ratio and inhibiting the growth of bacterial species *Erysipelotrichaceae, Bacillus* and *Eubacterium cylindroides*, which correlated with HFD-induced obesity (Etxeberria et al., 2015a). Moreover, it was shown in a recent study that curcumin, the major polyphenolic ingredient of an edible herb *Curcuma longa* L. improved intestinal barrier function by modulation of intracellular signaling, and organization of tight junctions, providing a mechanism that curcumin modulates chronic inflammatory diseases despite poor bioavailability (Wang J. et al., 2017). The details of some other herbal medicines, including formulae, herbals and phytochemicals reportedly to achieve their therapeutic effects for CMDs through gut microbiota modulation are summarized in Tables 1–3.

## Functional food and microbiota

Functional food has the advantages of wide availability, ease of preparation and fewer adverse effects. They could be well suited for CMDs remedies due to their potential effects such as anti-inflammatory, antioxidants, antiestrogenics, immunomodulatory, whereas purified active compounds are preferable as pharmaceutical drugs for the treatment of severe chronic symptoms (Martel et al., 2016; Meyer and Bennett, 2016). Epidemiological studies have identified associations between frequent consumption of fruits, vegetables, whole grains and teas, which are rich in fiber, polyphenol, and polysaccharide could reduce the risk of CMDs (Woodside et al., 2013; Klinder et al., 2016). These phytochemicals and their metabolic products may inhibit pathogenic bacteria while stimulating the growth of beneficial bacteria for CMDs (Laparra and Sanz, 2010).

Apples are among the most frequently consumed fruits to prevent obestiy by modulating gut microbiota with their multiple components, including fiber, pectin (Jiang et al., 2016), procyanidins (Masumoto et al., 2016) and polysaccharides (Wang S. et al., 2017). Administration with apple procyanidins (a subclass of polyphenols) for 20 weeks was able to reduce obesity, decrease lipid metabolism related genes expression, lower LPS levels and gut permeabiliby through decreasing the Firmicutes/Bacteroidetes ratio and increasing *Akkermansia* proportion (Masumoto et al., 2016). In addition, treatment with apple polysaccharide inhibited chronic inflammation, gut permeability, and SCFAs production, leading to lower abundance of Firmicutes and *Fusobacteium*, and higher *Bacteroidetes* and *Lactobacillus* in HFD-fed rats (Wang S. et al., 2017). Furthermore, the reciprocity between apple ingredients and the gut microbioa may benefit cardiovascular health (Koutsos et al., 2015). Unexpectedly, diet apple fiber and flavone were positively associated with *Blautia, Lactobacillus, Bifidobacterium*, and *Faecalibacterium*, showing great significance for the patients who suffer from systemic lupus erythematosus (SLE) (Cuervo et al., 2015; Table 4). As another example, ingestion of laminarin, a kind of polysaccharides extracted from *Laminaria* japonica, by HFD-fed mice significantly increased genus *Bacteroides* and decreased Firmicutes, with elevated energy metabolism (Nguyen et al., 2016; Table 5). Besides, 3,3-dimethyl-1-butanol (DMB), the structural analog of choline detected in some functional food such as balsamic vinegars, red wines, and olive oils (Kitai and Tang, 2017), is an inhibitor of TMA formation through inhibition of microbial TMA lyases. Therefore, it inhibited choline diet-promoted macrophage foam cell formation and atherosclerotic lesion development without altering the circulating cholesterol levels (Wang Z. et al., 2015).

**Table 4:** Functional food and gut microbiota.

**Functional Food**	**Ingredients**	**Objects**	**Diseases**	**Physiological function related to gut microbiota**	**Gut microbiota**	**References**
Vegetable/fruit juice	Polyphenols, Oligosaccharides, Fber, Nitrate	Twenty adults	Obesity	➀Alter the intestinal microbiota associated with weight loss, ➁Increase in vasodilator NO, ➂Decrease in lipid oxidation.	**Increased:** Bacteroidetes, Cyanobacteria, **Decreased:** Firmicutes, Proteobacteria.	Henning et al., 2017
Barley	β-Glucan	30 volunteers	CVD	N/A	**Increased:** Bacteroidetes, *Prevotella*, **Decreased:** Firmicutes, *Dorea*.	Wang Y. et al., 2016
Apple	(1) procyanidin	(1) C57BL/6J mice	(1) Obesity	(1) ➀Attenuate inflammatory effects and weight gain including gut permeability and lipopolysaccharide, ➁Decrease endogenous metabolites levels related with insulin resistance.	(1) **Increased:** *Akkermansia*, **Decreased:** Firmicutes/Bacteroidetes ratio.	Cuervo et al., 2015; Jiang et al., 2016; Masumoto et al., 2016; Wang S. et al., 2017
	(2) pectin	(2) SD rat	(2) Obesity	(2) ➀Attenuate weight gain and serum total cholesterol Level, ➁Improve intestinal alkalinephosphatase, claudin 1 expression, decrease *TLR4* expression in ileal tissue, decrease inflammation (TNF-α) and metabolic endotoxemia.	(2) **Increased**: Bacteroidetes, **Decreased:** Firmicutes.	
	(3) Polyphenols	(3) 20 Systemic lupus erythematosus patients	(3) Systemic lupus erythematosus	(3) N/A	(3) **Increased:** *Bifidobacterium*.	
	(4) Polysaccharide	(4) SD rat	(4) Microbial dysbiosis and chronic inflammation	(4) ➀Increase total SCFAs level, ➁Alleviate gut permeability and chronic inflammation (decrease LBP, up-regulation of occludin, down-regulation TNF, MCP-1, CXCL-1, IL-1β).	(4) **Increased:** Bacteroidetes, *Lactobacillus*, **Decreased:** Firmicutes, *Fusobacteium*.	
Oranges	Polyphenols	20 Systemic lupuserythematosus patients	Systemic lupus erythematosus	N/A	**Increased:** *Lactobacillus*,	Cuervo et al., 2015
Grape	(1) Pomace, Polyphenols	(1) Lamb	(1) N/A	(1) Decrease oxidative stress-induced damage to lipids and proteins such as TBARS and CARB.	(1) **Decreased:** *Enterobacteriacae*, *Escherichia coli*.	Baldwin et al., 2016; Kafantaris et al., 2016
	(2) N/A	(2) C57BL/6J mice	(2) Obesity	(2) ➀Decrease triglyceride and liver weight levels and reduce *GPAT1* expression, ➁Reduce hepatic mRNA *PPAR-γ2, SCD1, FABP4* and *GPAT1* levels	(2) **Increased:** *Akkermansia muciniphila, Allobaculum*, **Decreased:** *Desulfobacter* spp.	
Grape seed	Proanthocyanidin	C57BL/6 mice	Obesity	➀Decrease plasma inflammatory factors TNF-α, IL-6 and MCP-1 levels, ➁Ameliorate macrophage infiltration, ➂Reduced epidydimal fat mass and improve insulin sensitivity.	**Increased:** *Clostridium XIVa*, *Roseburia*, *Prevotella*.	Liu et al., 2017
Agave salmiana	Saponin	C57BL/6 mice	Obesity and hepatic steatosis	➀Reduce fat mass and weight gain, Lower insulin, glucose, and LDL levels, ➁Lower hepatic lipid levels and HOMA index, increase fatty acid oxidation related genes expression, ➂Increase fatty acid oxidation, AMPK phosphorylation, white adipose tissue browning, mitochondrial activity and energy expenditure.	**Increased:** *Akkermansia muciniphila*.	Leal-Diaz et al., 2016
Cranberry	Cranberry extract	C57BL/6J mice	Obesity	➀Decrease liver weight and triglyceride accumulation involved in inflammation and blunted hepatic oxidative stress, ➁Improve insulin tolerance, decrease glucose-induced hyperinsulinaemia, ➂Lower intestinal triglyceride content and alleviate intestinal oxidative stress and inflammation.	**Increased:** *Akkermansia*.	Anhe et al., 2015
Bamboo shoot	Fiber	C57BL/6J mice	Obesity	Lose weight.	**Increased:** Bacteroidetes, **Decreased:** Verrucomicrobia.	Li et al., 2016
Nopal	Fiber, Polyphenols, Vitamin C	Wistar rat	Obesity	➀Modify gut microbiota and increase intestinal occludin-1, ➁Decrease in LPS, glucose insulinotropic peptide, glucose intolerance, lipogenesis, and metabolic inflexibility, ➂Reduce hepatic steatosis and oxidative stress in adipose tissue and brain, ➃Improve cognitive function.	**Increased:** Bacteroidetes/Firmicutes ratio, *Anaeroplasma*, *Prevotella*, *Ruminucoccus*, *Bacteroides fragilis*, **Decreased:** *Faecalibacterium*, *Clostridium*, *Butyricicoccus*.	Sanchez-Tapia et al., 2017
Wheat	(1) Enzyme-treated wheat bran,	(1) C57BL/6J mice	(1) Obesity	(1) ➀Decrease body weight and liver TGs, increase index of liver reactive oxygen species, ➁Decrease liver antioxidants (glutathione and α-tocopherol) and liver carbohydrate metabolites (glucose); lower hepatic arachidonic acid; and increase liver and plasma β-hydro*xyb*utyrate.	(1) **Increased**: Bacteroidetes, **Decreased:** Firmicutes.	Neyrinck et al., 2011; Kieffer et al., 2016
	(2) Arabinoxylan	(2) C57BL/6J mice	(2) Obesity	(2) ➀Regulate host metabolic parameters: reduce body weight gain, fat mass development, inflammation (serum IL-6, MCP-1), cholesterolemia and insulin resistance, and increase gut junction proteins. ➁Regulate host adipose tissue: reduce lipogenesis (Fatty acid synthase), fatty acid oxidation (carnitinepalmitoyl transferase-1), fatty acid uptake (lipoprotein lipase) and GPR-43 expression, and increase adipocyte area and rumenic acid.	(2) **Increased:** *Bacteroides*/*Prevotella* ratio, *Bifidobacteria*, *Roseburia* spp.	
Oat	N/A	SD rat	Obesity	➀Decrease body weight, epididymal fat accumulation, and serum inflammatory factor (TNF-α) levels and significantly regulate serum lipid levels, ➁Increase the total SCFA concentration in colonic digesta.	**Increased:** Bacteroidetes, Bacteroidetes/Firmicutes Ratio, **Decreased:** Firmicutes.	Dong et al., 2016
Tea Polyphenols	Polyphenols	C57BL/6 ApoE–/– mice	AS	➀Decrease the total cholesterol and low-density lipoproteincholesterol, ➁Decrease the plaque area/lumen area ratios.	**Increased:** *Bifidobacteria*.	Liao et al., 2016
Green tea and isomalto-oligosaccharides	N/A	HFD-induced male Swiss albino mice	Obesity	Prevent leaky gut phenotype and LPS, pro-inflammatory cytokines (*e.g*. resistin, adiponectin, TNF-α, IL-1β, IL-6) increase	**Increased:** *Lactobacillus*, *Bifidobacteria*, *Akkermansia*, *Roseburia* spp., *Prevotella/Bacteroides*, **Decreased:** Firmicutes/Bacteriodetes ratio.	Singh et al., 2017
Yellow pea	Fiber	SD rat	Obesity	Lower final percent body fat.	**Decreased:** Firmicutes.	Eslinger et al., 2014
Green tea, oolong tea, black tea	8 phenolic acids, 12 flavanols, 9 flavonols, 2 alkaloids, 1 amino acids	C57BL/6J mice	Obesity	Trend to lose weight.	**Increased:** *Alistipes*, *Rikenella*, *Lachnospiraceae*, *Akkermansia*, *Bacteroides*, *Allobaculum*, *Parabacteroides*.	Liu et al., 2016
Fuzhuan tea	N/A	Wistar rats	NAFLD	Reduce plasma leptin and prevent high saturated fat diet-induced inflammation.	**Increased:** *Lactobacillus* spp.	Foster et al., 2016

**Table 5:** Functional food phytochemicals and gut microbiota.

**Phytochemicals**	**Category**	**Objects**	**Diseases**	**Physiological function related to gut microbiota**	**Gut microbiota**	**References**
Laminarin	Polysaccharide	BALB/c mice	Obesity	Reduce energy metabolism	**Increased:** Bacteroidetes (especially the genus *Bacteroides*), **Decreased:** Firmicutes.	Ko et al., 2014; Nguyen et al., 2016
Fucoidan	Polysaccharide	C57BL/6J mice	Intestinal dysbiosis	Reduce inflammatory response and antigen load, and decrease LPS-binding protein levels.	**Increased:** *Lactobacillus*, *Ruminococcaceae*, **Decreased:** *Peptococcus*.	Shang et al., 2016
Melatonine	Alkaloid	C57BL/6J mice	Obesity	Change gut microbiota composition	**Increased:** *Akkermansia*, **Decreased:** Firmicutes/Bacteroidetes ratio.	Xu P. et al., 2017
Fructans	Polysaccharide	C57BL/6J mice	Obesity	Improve intestinal physiology and shift gut microbiota.	**Increased:** Actinobacteria, Verrucomicrobia (*Akkermansia*).	Liu J. P. et al., 2016
Capsaicin (Chili peppers)	Alkaloid	C57BL/6J mice	Obesity	➀Prevent HFD-induced gut barrier dysfunction by inhibiting cannabinoid receptor type 1 (CB1). ➁Protect against HFD-induced obesity is transferrable.	**Increased:** Ruminococcaceae, Lachnospiraceae.	Kang et al., 2017
3,3-dimethyl-1-butanol (DMB)	Choline analogue	C57BL/6J mice	AS	Reduce microbial trimethylamine formation and inhibit choline diet-enhanced AS.	N/A	Wang Q. et al., 2015

Moreover, vegetables (e.g., bamboo shoot), whole grains (e.g., wheat, barley and oat), and teas (e.g., green tea, oolong tea, black tea and fuzhuan tea), exert positive effects on CMDs through modulating gut microbiota (Table 4). Numerous instances are detailed in Tables 4, 5. Although, intervention studies conducted both in animals and humans have demonstrated beneficial effects of functonal foods on anti-inflammation, vascular function, and energy metabolism, the apparent association with altered gut microbiota is still lacking.

## Overlaping effects between herbal medicine and functional food on gut microbiota

Medicine and food deriving from the same source has been realized since ancient times. In TCM, food is conceptualized according to both nutritional and functional aspects, and can be used to treat illnesses. The “medicine-food homology” concept has given a new meaning since the discovery of human-microbiota existing as a whole symbiotic ecosystem. Interestingly, a series of overlapping characteristics through modulating gut microbiota for CMDs between herbal medicine and functional food are uncovered based on the description above (Tables 1–5): ➀shared components, ➁similar functions, ➂common mechanisms, and ➃same intestinal microbiota.

First and foremost, there is no absolute boundary between medicine and food. Some medicines are food whereas certain foods can be employed as medicine. *Lonicera japonica Thunb, Cassia obtusifolia L., Semen Coicis*, adlay, *Zingiber officinale Roscoe* (major ingredient: curcumin), and mulberry (main ingredient: RSV), are edible medicines, there are only dosage differences between edible and medicinal use. These herbs not only belong to medicine with valid efficacies for CMDs remedy, but also are delicious food with rich nutrients. Besides, some medicines have been developed into nutraceuticals, which contain important natural bioactive compounds that confer health-promoting and medical benefits to humans, such as *Ganoderma lucidum, Herba Epimdii, Ophiopogon japonicas, Rehmannia glutinosa Libosch, Rheum rhabarbarum* (major ingredient: rhein), and lingonberry, see Tables 1–3. Furthermore, many foods could serve as nutraceutical candidates, and some of those, such as pomegranate peel, bamboo shoot, grape (major ingredient: RSV), and laminarin have the potential to branch into medicines (Tables 4, 5).

Secondly, the components of fibers (e.g., bamboo shoot, nopal, and yellow pea), polyphenols (e.g., GQD, *Flos Lonicera*, adlay, apple, grape, orange, nopal and tea) and/or polysaccharides (e.g., Ginseng decoction, Yupingfeng, *Ganoderma lucidum, Polygonatum kingianum, Ophiopogon japonicas*, apple and barley) are shared in most herbals and foods, exerting prebiotics-like effects for CMDs, which can be seen in Tables 1–5. Moreover, polyphenol phytochemicals such as RSV and quercetin are present in both herbals and foods. These components are able to escape absorption in the upper gastrointestinal tract and reach the large intestine without breaking down. Thus, these components can also be converted by local microbiota to biologically active and bioavailable metabolites with systemic effects.

Thirdly, just as what we have introduced above, gut microbiota-derived metabolites such as SCFAs, LPS, Bas, and TMAO are the most likely microbial metabolites linking CMDs remedy to intestinal microbiota. Numerous herbals and foods are likely to prevent and treat CMDs through these mediators. Improved gut permeability and gut integrity in conjunction with the increased expression of ZO-1 and/or occludin-1 and/or claudin-1, resulted in reduction of circulating LPS levels and a series of inflammatory response, which are affected by herbals (Yupingfeng polysaccharides enhanced immunity, Sun H. et al., 2016). QHD was used in the treatment of NAFLD (Feng et al., 2017); *Flos Lonicera* ameliorated obesity (Wang J. H. et al., 2014); curcumin attenuated AS (Ghosh et al., 2014) and foods [apple derived polyphenols and polysaccharide prevented obesity, (Masumoto et al., 2016; Wang S. et al., 2017), nopal and capsaicin were used in combacting obesity (Kang et al., 2017; Sanchez-Tapia et al., 2017)]. SCFAs production was shown to restore aberrant levels of gut hormones such as GLP-1, PYY, and the activation of GPR43. SCFAs production are also promoted by berberine in energy metabolism, insulin resistance and obesity (Xie et al., 2011; Zhang et al., 2015; Xu J. H. et al., 2017); elevated by apple polysaccharide in chronic inflammation, and enriched by oat in obesity treatment (Wang S. et al., 2017). In addition, *Polygonatum kingianum* and taurine intervene with both SCFAs and LPS levels in different CMDs (Yu et al., 2016; Yan et al., 2017). TMAO levels were inhibited via the reduction of TMA formation by RSV and then attenuate AS (Wang Z. et al., 2015; Chen M. L. et al., 2016). At the same time, RSV increased BAs deconjugation and fecal excretion by enhancing the activity of hydrolase activity, which displayed correlation with the lowered BA content in ilealby suppressing FXR-FGF15 axis and promoting *CYP7A1* expression (Chen M. L. et al., 2016). All of these interventions are along with the changed microbiota composition. An increasing number of metabolic pathway and potential mechanisms are studied on the mediators of SCFAs, LPS, BAs and TMAO. These studies provide a better understanding of how herbals and foods prevent or treat CMDs by gut microbiota. The cross-talk between these mediators and specific alteration of intestinal bacteria in host physiology, as well as the precise contributing elements in herbals and foods for CMDs remedy shoud be subjects for future studies.

Finally, previous work has established that genera *Clostridium, Lactobacillus* and *Ruminococcus*, as well as the butyrate producers *Eubacterium, Fecalibacterium* and *Roseburia* are the important members of Firmicutes. Bacteroidetes including the genus *Bacteroides, Prevotella* and *Xylanibacter* are known to be efficient degraders of dietary fiber (Simpson and Campbell, 2015). Genus *Bifidobacterium* is a major member of Actinobacteria. Proteobacteria contains *Escherichia* and *Desulfovibrio*, whereas Verrucomicrobia includes only the mucus-degrading genus *Akkermansia* so far (Schroeder and Backhed, 2016). Tables 1–5 show that the ratio of Firmicutes/Bacteroidetes is modulated in most herbal- and food-intervention studies for CVD as well as various metabolic diseases. For example, decreased ratio of Firmicutes/Bacteroidetes was observed in obesity after intervened by herbals [Daesiho-tang (Hussain et al., 2016), *Ganoderma lucidum* (Chang C. J. et al., 2015), *Flos Lonicera* (Wang J. H. et al., 2014), *Rhizoma coptidis* (Xie et al., 2011), Resveratrol (Qiao et al., 2014), Berberine (Xie et al., 2011; Zhang et al., 2012a), Quercetin (Etxeberria et al., 2015b)] and foods [apple (Jiang et al., 2016; Masumoto et al., 2016), nopal (Sanchez-Tapia et al., 2017), wheat (Kieffer et al., 2016), Laminarin (Nguyen et al., 2016)], as well as in QHD (Feng et al., 2017) and berberine (Cao et al., 2016) for NAFLD (Feng et al., 2017), and barley for CVD (Wang Y. et al., 2016). All of these studies confirmed that increase in gut bacteria phylum Bacteroidetes, and inhibition of Firmicutes, and alteration of Firmicutes/Bacteroidetes ratio helped to treat CMDs including obesity (Ley et al., 2006; Sweeney and Morton, 2013), insulin resistance (Greenhill, 2015), NAFLD (Liu J. P. et al., 2016) and CVD (Marques et al., 2017). In addition, an increase in the *Akkermansia* population was found to be in favorable treatment for T2D (Shin et al., 2014), obesity (Everard et al., 2013), AS (Li J. et al., 2016) and some other metabolic syndromes (Roopchand et al., 2015). A recent study showed that fat mass development, insulin resistance and dyslipidemia were reduced by purified membrane protein from *Akkermansia* (Plovier et al., 2017). Interestingly, the abundance of *Akkermansia* was dramatically increased not only by Daesiho-tang for T2D (Hussain et al., 2016) and agave salmiana for hepatic steatosis (Leal-Diaz et al., 2016), but also by *Flos Lonicera* (Wang J. H. et al., 2014), pterostilbene (Etxeberria et al., 2016), apple (Masumoto et al., 2016), grape (Baldwin et al., 2016), agave salmiana (Leal-Diaz et al., 2016), cranberry (Anhe et al., 2015), green tea (Liu Z. et al., 2016; Singh et al., 2017), melatonine (Xu P. et al., 2017) and capsaicin (Kang et al., 2017) for obesity. Moreover, various diseases such as obesity, diabetes and ballergies have been associated with lower numbers of *Bifidobacterium* at various stages of life (Arboleya et al., 2016). The therapeutical effects of RS for AS (Chen M. L. et al., 2016), berberine for NAFLD (Cao et al., 2016), and *Rehmannia glutinosa Libosch* (Han et al., 2015), Daesiho-tang (Hussain et al., 2016), rhein (Wang et al., 2016), tea polyphenols (Singh et al., 2017), green tea (Singh et al., 2017) for obesity were associated with the elevated abundance of *Bifidobacterium*. What's more, increased abundance of genus *Lactobacillus, Bacteroides*, and *Prevotella* which contribute to metabolic diseases and/or CVD, were also closely associated with digestion of herbals and foods, as shown in Tables 1–5. In summary, increasing the abundance of phylum Bacteroidetes, and genus *Akkermansia, Bifidobacterium, Lactobacillus, Bacteroides*, and *Prevotella*, while reducing phylum Firmicutes and Firmicutes/Bacteroidetes ratio may serve as the common characteristics for gut bacteria modulation of herbal medicine and functional food for CMDs. For future studies, the related gut microbiota species interplay with plants and mammalian hosts need to be further investigated.

## Potential effects of herbal medicine and functional food on gut-organs axes

Commensal gut bacteria impact the host health especially CMDs processes in multiple organs. Several new concepts are proposed in recent reviews focusing on the relationship between gut and organs, such as gut-heart axis (Buglioni and Burnett, 2013), gut-brain axis (De Clercq et al., 2017; Dinan and Cryan, 2017), gut-liver axis (Wiest et al., 2017), gut-kidney axis (Katagiri et al., 2013; Budden et al., 2017) and gut-liver-lung axis (Young et al., 2016). The gut is no longer viewed as just a digestive organ, it is also considered as a metabolic and immunomodulatory organ. The major components of fiber, polyphenols and polysaccharides are present in large quantities in both herbal medicine and functianl food, which we have analyzed above. Besides, their muti-ingredient, muti-target and muti-pathway mode are well known and capable of meeting the complex system of the gut-organ interactions. Targetinig the gut-organs axes may also be responsible for CMD treatment. The potential effects are implicated by some latest reports. For instance, a recently published study found that chronic prebiotic treatment indeed exhibited both antidepressant and anxiolytic effects, reduced stress-induced corticosterone release, and modified specific gene expression in the hippocampus and hypothalamus. These effects were exerted via increased cecal acetate and propionate and reduced isobutyrate concentrations. These findings provided clear evidence supporting therapeutic targeting of the gut microbiota for gut-brain axis disorders (Burokas et al., 2017). Another recent finding (Marques et al., 2017) illustrated how HFD and supplementation with acetate influenced gut-heart-kidney axis in a mouse hypertension and heart failure model. It was found that both fiber and acetate decreased gut dysbiosis, measured by the ratio of Firmicutes to Bacteroidetes, and increased the prevalence of Bacteroides acidifaciens. Both HFD and acetate supplementation significantly reduced blood pressure, cardiac fibrosis, and left ventricular hypertrophy. Transcriptome analyses showed that the protective effects of high fiber and acetate were accompanied by the downregulation of cardiac and renal early growth response protein 1 (EGR1), a master cardiovascular regulator involved in cardiac hypertrophy, cardiorenal fibrosis, and inflammation. The upregulation of a network of genes involved in circadian rhythm in both tissues and downregulation of the renin-angiotensin system in the kidney and MAPK signaling in the heart presents an interesting example of gut-multi organs interactions that are simultaneously affected by diet via microbiota.

Althrough there are no reports on herbal medicine or functional food directly targeting gut-organs axes, more study should be carried out in the area to fully exploit the beneficial aspects of gut microbiota. Major components such as fiber and polysaccharide could be fermented and converted into SCFAs which has been shown to be beneficial on the treatment of CMDs. It is foreseeable that the influence of functional food and herbal medicine on the interactive and dynamic relationships between gut microbiota and essential organs will be elucidated in the future.

## Conclusions and perspective

The concept of “medicine-food homology” has evolved from its ancient origin and is given a new prospective with newly revealed role of gut microbiota of the host. Human diseases, particularly CMDs, not only could be treated by herbal derived medicine, but also could be prevented by medicinal food via co-inhabiting and influcing gut microbiota.

**Figure 1:**
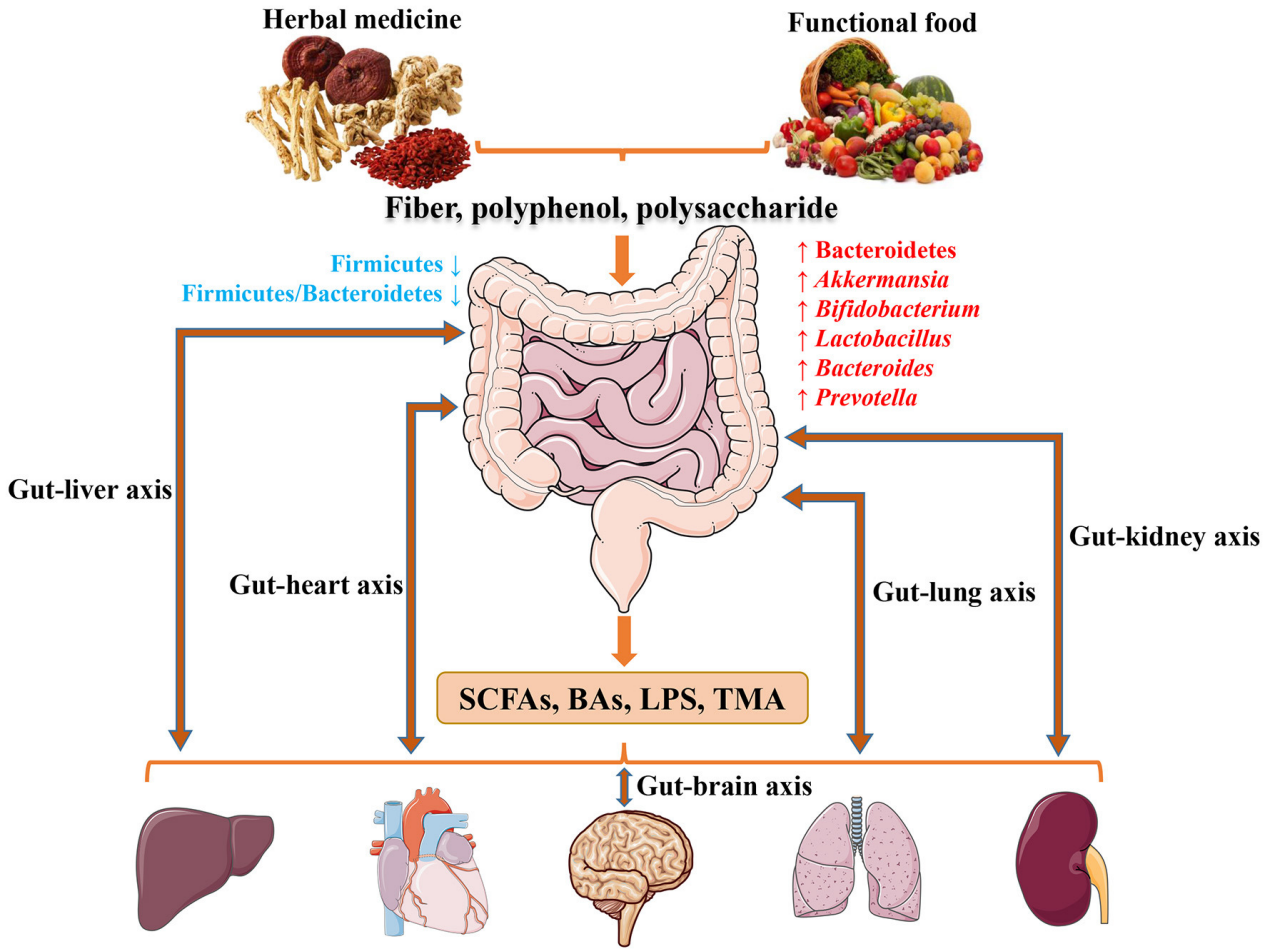
The potentially shared biological processes and underlying mechanisms of herbal medicine and functional food for CMDs through modulating microbiota.

With the rapid advancement of sequencing technology and intense efforts by researchers, a significant understanding of host gut microbiota has been achieved (Xiao et al., 2017). It is also becoming apparent that herbal medicine and functional food may strongly influence gut microbiota associated with CMDs in humans ranging from obesity to T2D and CVD. Nevertheless, studies on the interaction between herbal derived bioactive compounds and gut microbes are still needed. Future investigation in the field may include, but not limited to the following directions: (1) disease-related and disease-specific microbiota and pathological mechanisms; (2) future research on herbal medicine and functional food should exploit molecular mechanisms and the relationship between microbiota and host behavior; (3) until now, most medicine and functional food research has focused on obesity and T2D rather than CVD, which deserves more careful studies and funding; (4) certain polyphenols (puerarin, paeoniflorin, baicalein, icariin, mangiferin, gallic acid, luteolin, cryptotanshinone, kaempferol, etc.) are similar to RSV and showed poor absorption into the bloodstream after oral administration, but they may have an impact on gut microbiota as well. In conclusion (Figure 1), herbal medicine and functional food with major ingredients including fiber, polyphenols and polysaccharides are inclined to increasing abundance of phylum Bacteroidetes, and genus *Akkermansia, Bifidobacterium, Lactobacillus, Bacteroides*, and *Prevotella*, while reducing phylum Firmicutes and Firmicutes/Bacteroidetes ratio to prevent or treat CMDs through SCFAs, BAs, LPS and TMAO signaling. The condition of health or disease in human is critically dependent on the balance between medicine/food by modulating gut microbiota. Human intake of herbal medicine and functional food can alter gut microbiota, and microbiome in turn can influce human health through microbial metabolites. The convergence between herbal medicine and functional food through microbiota reinforces the idea that CMDs are not only treatable but also preventable by maintaining the balance between the two.

## Author contributions

YZ conceived, designed, organizedand revised the manuscript; ML conceived, designed, wrote and revised the manuscript; YW, GF, XW, and SX revised the manuscript and discussed interpretation.

### Conflict of interest statement

The authors declare that the research was conducted in the absence of any commercial or financial relationships that could be construed as a potential conflict of interest.

## Abbreviations

CMDs: Cardiometabolic diseases
CVD: cardiovascular disease
T2D: type 2 diabetes
NAFLD: non-alcoholic fatty liver disease
AS: atherosclerosis
CKD: chronic kidney disease
MI: myocardial infarction
SCFAs: short-chain fatty acids
Bas: bile acids
LPS: lipopolysaccharide
TMAO: trimethylamine–*N*-oxide
TCM: traditional Chinese medicine
MetaHIT: Metagenomics of the Human Intestinal Tract
GI: gastrointestinal
HFD: high-fat-diet
UC: ulcerative colitis
AMR: antimicrobial resistance
RSV: resveratrol
GQD: GegenQinlian Decoction
FMO3: flavin-containing monooxygenase 3
GLP-1: glucagon-like peptide-1
MCP1: monocyte chemoattractant protein 1
ZO-1: zona occludens-1
FXR: farnesoid X receptor
FGF15: fibroblast growth factor 15
Fiaf: fasting-induced adipose factor
TLR4: toll-like receptor 4
GPAT1: glycerol-3-phosphate acyltransferase 1.
